# Assessment of European and hybrid aspen clones efficiency based on height growth and removal percentage of petroleum hydrocarbons—a field trial

**DOI:** 10.1007/s11356-020-10453-4

**Published:** 2020-08-15

**Authors:** Mir Md Abdus Salam, Muhammad Mohsin, Fahad Rasheed, Muhammad Ramzan, Zikria Zafar, Pertti Pulkkinen

**Affiliations:** 1 School of Forest Sciences, University of Eastern Finland, Yliopistokatu 7, P.O. Box 111, 80100 Joensuu, Finland; 2 Department of Forestry & Range Management, University of Agriculture, Faisalabad, 38000 Pakistan; 3 College of Forestry, Nanjing Forestry University, Nanjing, 210037 China; 4 Department of Soil and Water Conservation and Desertification, Beijing Forestry University, Beijing, 100083 China; 5 Natural Resources Institute Finland (Luke), Haapastensyrjä Research Unit, Haapastensyrjäntie 34, 12600 Layliainen, Finland

**Keywords:** European aspen, Hybrid aspen, Hydrocarbons, Phytoremediation

## Abstract

Soils polluted by organic or inorganic pollutants are an emerging global environmental issue due to their toxic effects. A phytoremediation experiment was conducted to evaluate the extraction potential of three European aspen clones (R2, R3, and R4) and seven hybrid aspen clones (14, 27, 34, 134, 172, 191, and 291) grown in soils polluted with hydrocarbons (includes polycyclic aromatic hydrocarbons (PAH) and total petroleum hydrocarbons (TPH)). Height growth, plant survival rates, and .hydrocarbon removal efficiencies were investigated over a 4-year period at a site in Somerharju, Luumaki Finland, to assess the remediation potential of the clones. Hydrocarbon content in the soil was determined by gas chromatography and mass spectrometry. The results revealed that hybrid aspen clones 14 and 34 and European aspen clone R3 achieved greater height growth (171, 171, and 114 cm, respectively) than the other clones in the study. Further, the greatest removals of PAH (90% at depth 10–50 cm) and (86% at depth 5–10 cm) were observed in plot G15 planted with clone R2. Furthermore, the greatest TPH removal rate at 5–10 cm depth (C_22_–C_40_, 97%; C_10_–C_40_, 96%; and C_10_–C_21_, 90%) was observed in plot 117 with clone 134. However, other clones demonstrated an ability to grow in soils with elevated levels of TPH and PAH, which indicates their tolerance to hydrocarbons and their potential capacity for phytoremediation of hydrocarbon-polluted soils. Our study suggests that European aspen and hybrid aspen clones could be used for the remediation of soils polluted with PAH and TPH.

## Introduction

Globally, petroleum is considered an essential component of modern industrial society (Khan et al. 2013). Unfortunately, sites are contaminated with petroleum hydrocarbons due to the processing of petroleum derivatives through extraction, transportation, refining, and usage (PAH; Khan et al. 2013; Nam et al. 2008). Further, petroleum-based products are a known energy source for daily life and industrial performance, although the discharge of these products into the environment results in ecosystem damage and genetic mutation, such as pollutant deposition in plant tissues and animals (Guarino et al. 2017), as well as negatively influences plant growth and development (Nie et al. 2011). Moreover, the occurrence of petroleum pollutants in the soil strongly affects soil chemical properties, microbial populations, and performance (Guo et al. 2012; Leme et al. 2012) and also negatively impacts the capacity of plants and microbes to absorb water and nutrients from the soil (Kathi and Khan 2011). Crude oil is a complex combination of hydrocarbon and non-hydrocarbon compounds and has diverse effects on soil microorganisms, with the discharge of crude oil into the soil known to alter both the physical and chemical properties (Udeh et al. 2013).

The complex organic chemicals include PAH, which are enclosed by carbon and hydrogen and have a melded ring structure, partially holding two benzene rings (Ravindra et al. 2008). In general, PAH are produced through wood and coal combustion, diesel and petrol combustion, and industrial processes and can be found in the surrounding environment, including the air, water, and soil (Semenov et al. 2017). Emissions of PAH into the soil may eventually cause fires or dangerous explosions, especially when the fumes enter restrained places (Souza et al. 2014). Furthermore, PAH are also spread naturally through forest fires and volcanic eruptions and are considered an incomplete product that is formed by the pyrolysis of fossil fuels (Wcisło 1998).

The presence of total petroleum hydrocarbons (TPH) in the soil hinders aeration, since the oil fumes produce a physical obstacle between the air and the soil, thus resulting in distortion of the soil texture. Hence, these detrimental effects lead to changes in soil redox potential, which leads to the increases in soil pH and the suffocation and toxicity of soil biota (Olawepo et al. 2018). Indeed, TPH are considered environmental stressors that can cover the plant root surface and prevent water and nutrient uptake. Moreover, they can enhance the production of reactive oxygen species (ROS), leading to the activation of oxidative stress in plants (Noori et al. 2018).

Globally, an average of 35 million barrels of petroleum is transported across the seas and oceans every year, and this renders the marine ecosystem susceptible to contamination (Macaulay 2015). It has been estimated by the European Environmental Agency (EEA) that there are approximately 2.5 million potentially polluted sites in Europe (EEA 33 plus the six cooperating countries). Heavy metals (HM) and petroleum products are the fundamental causes of soil contamination (Cocârţă et al. 2017). Also, crude oil and petroleum elements, such as PAH, volatile aromatic hydrocarbons (VAH), benzene, toluene, ethylbenzene, and xylenes (BTEX), have affected up to approximately 53% of the polluted sites in Europe (Pinedo et al. 2013). Moreover, about 45% of groundwater in Europe is contaminated with crude oil and petroleum elements. Groundwater contamination with PAH and BTEX has increased from 6 to 15 % (Panagos et al. 2013). In the European environment, PAH accounts for up to 13% of the pollutants that affect soils (due to their low water solubility and low volatility) (Istrate et al. 2018) and can be found in the soil at levels as high as 3330 mg kg^–1^ (Kołtowski et al. 2016).

In Finland, PAH, TPH, polychlorinated biphenyls, chlorophenols, and pesticides, including HM, such as arsenic (As), lead (Pb), copper (Cu) and zinc (Zn), are considered significant sources of soil pollution. These pollutants originate from the dispersion and storage of fuel-impregnated plants, diverse industrial activities, depots and garages, greenhouses, and shooting ranges (Kuusiniemi and Eklund 2008; Mohsin et al. 2019; Paccassoni et al. 2017). Moreover, the long-term persistence of inorganic pollutants in the soil causes considerable stress to forest ecosystems in the region, which then require extensive remediation (Xu 2013). However, a cost-effective biological technology, such as phytoremediation (e.g., the use of specific plants and/or their rhizosphere microorganisms to decrease, destroy, and alleviate the toxic pollutants), is required to efficiently overcome the problems associated with environmental pollution with minimal ecological impact and neutralize HM- and PAH-polluted soils (Zalesny et al. 2005).

In recent years, the emerging innovative technologies for the removal of organic or inorganic pollutants have been widely explored. Formerly, prevalent remediation methods, such as physical remediation, chemical remediation, and bioremediation, have been utilized to rehabilitate polluted soils, but these methods are considered costly and likely to induce further contamination (Peng et al. 2009; Salam et al. 2019). In this context, phytoremediation has been seen as an attractive, eco-friendly, cost-efficient, and economically sustainable method to remove pollutants, using specific metal tolerant plants (Mohsin 2016; Salam et al. 2016). Phytoremediation of hydrocarbon-polluted soils is commonly driven by the process called rhizosphere degradation, in which the increased microbial activity associated with the soil, in proximity to plant roots, breaks down the hydrocarbons (Tischer and Hübner 2002).

*Populus* is a genus of 25–35 species of deciduous plants in the family of *Salicaceae*, which are native to the Northern Hemisphere. The genus has considerable genetic heterogeneity and can grow up to 50 m (Zacchini et al. 2011). The European aspen (*Populus tremula* L.) has been considered a suitable candidate for phytoremediation of soils polluted with organic and inorganic pollutants due to inherent characteristics, such as abundant genetic variation in a natural population, rapid physiological responses to environmental factors, well-characterized molecular physiology, cloning of individual tree genotype, as well as exhibiting considerable biomass production rates, a high transpiration rate, and an extensive root system (Guerra et al. 2011). The European aspen tolerates a wide range of extreme climatic and soil conditions and has been used for phytoremediation in the past due to its rapid growth and considerable biomass production rates, ability to grow in a polluted environment, as well as its capacity to remove HM and TPH from soils (Malá et al. 2007; Mukherjee 2014). Hybrid aspen (*P. tremula* L*.* × *P. tremuloides* Michx.) is a cross between the European aspen and North American trembling aspen (*P. tremuloides* Michx.), is one of the fastest-growing tree species in Finland, and has been widely used for phytoremediation and for biomass production for energy generation (Häikiö et al. 2009; Tullus et al. 2012).

Between 1947 and 1958, the Somerharju area in Southeast Finland was a creosote preservation facility for railway sleepers (Clavel 2013). Creosote oil is a by-product of tar distillation, which contains around 85% PAH, 15% TPH, and high concentrations of C_10_–C_21_, C_22_–C_40_, C_10_–C_40_, phenanthrene, and naphthalene (Mueller et al. 1989). In general, 20–40% of creosote is comprised of sixteen (US EPA-defined) priority PAH pollutants (Kulik et al. 2006). The soil in this area is polluted with sixteen priority PAH (previously reported by Golder Associate Corporation, Helsinki) and has become a threat to groundwater and ecosystems. As the detrimental effects of oil spillage on soils are of considerable global concern (Khan et al. 2016; Obida et al. 2018), this study would help create awareness of the potential of European aspen and hybrid aspen clones for the remediation of polluted soils in Finland, as well as in other parts of the world.

To the best of our knowledge, this field trial is the first attempt to evaluate the phytoremediation efficiency of divergent European aspen and hybrid aspen clones to remediate hydrocarbon-polluted soils at a former railway sleeper site. Principally, this study aimed to assess the growth and phytoremediation efficacy of European aspen and hybrid aspen clones in soils polluted with PAH and TPH, as well as identify potential European aspen and hybrid aspen clones that could be used to reduce PAH and TPH concentrations in polluted soils.

## Materials and methods

### Study site and planting materials

The experimental site “Somerharju” is located in southeastern Finland (60° 55′ 00″ N and 27° 26′ 00″ E, Luumaki). During the growing season, the average monthly air temperature varies in the study area, typically from 25 °C in the summer to – 22 °C in the winter. Daylight varies from 5 h in winter to 20 h in summer. Based on breeding experiments (cloned by micropropagation in the Haapastensyrja Unit of Natural Resources Institute Finland (Luke)), three European aspen (*P. tremula)* clones, R2, R3, and R4, and seven hybrid aspen (*P. tremuloides Michx.* × *P. tremula* L.) clones, 14, 27, 34, 134,172, 191, and 291, were selected for the study.

### Experimental design

**Fig. 1:**
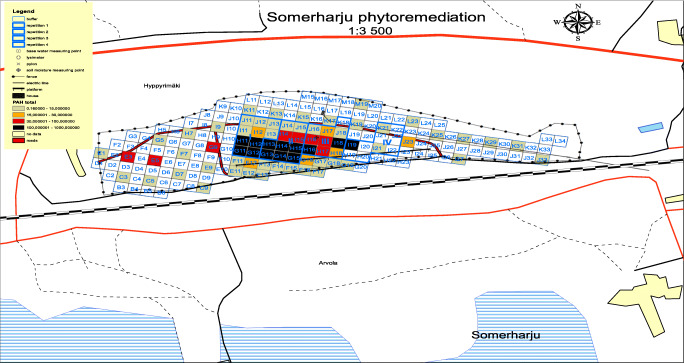
Map of the Somerharju experimental site

The total study area was ~ 6.7 ha, with 2 ha highly polluted with hydrocarbons. We prepared a total of 40 square plots (20 × 20 m) for the planting of European aspen and hybrid aspen clones. Before the preparation of the plots, we clear cut all the other plants in the polluted area (~ 2 ha). Based on the pollutant concentrations, the 40 square plots were assigned to one of four blocks (I–IV). Each plot was further divided into 400 smaller sub-plots (area 1 m^2^). Next, 2-year-old European aspen or hybrid aspen trees were planted in each sub-plot at a planting density of 10,000 trees/ha (Fig. 1). All clones were planted in repetition one, except for clone 34, because that plot had an extraordinarily high density of European aspen. Tap water was used to irrigate the plants, and some plots (e.g., H13, H14, and H15) (Fig. 1) planted with clones 134, R3, and 291, respectively, were fertilized with NPK, to examine the effect of fertilizer application on plant growth.

### Growth measurements

Height growth (cm) of the European aspen and hybrid aspen clones, which grew for a 4-year period (2013–2017), was measured as the distance from the ground to the tallest living bud point.

### Soil sampling and soil characteristics

Soil samples were collected in 2011 from the study area and were observed to be polluted with PAH, comprising sixteen priority pollutants (e.g., naphthalene, acenaphthene, acenaphthylene fluorene, phenanthrene, anthracene, fluoranthene, pyrene, benzo(a)anthracene, chrysene, benzo(b)fluoranthene, benzo(k)fluoranthene, benzo(a)pyrene, indeno(1,2,3–cd)pyrene, benzo(ghi)perylene, dibenzo(a, h)anthracene, and TPH (C_10_–C_21,_ C_10_–C_40_, and C_22_–C_40_). The area was sampled using a 20 × 20 m grid, and sampling pits were excavated in each plot to a depth of 5–10 cm or 10–50 cm depths with a shovel. Five samples were taken from each plot, which included one sample from the center of the pit and the remaining four samples from the corners of the square plot. Approximately 0.25 L of soil was collected from each pit, mixed, and combined as a 1-L composite sample in an airtight sampling bag. The composite sample was sealed in the bag, and soil samples were then stored in cold storage for 1–2 days, until the samples were sent to the laboratory for chemical analysis.

The basic properties of the soil from different polluted plots (Mean ± SE) were previously determined by Mukherjee et al. (2014): pH, 5–8; available N, 0.025 ± 0.012 mg/kg; available P, 207 ± 36 mg/kg; available K, 510 ± 67 mg/kg; available Ca, 1272 ± 409 mg/kg; available Mg, 613 ± 107 mg/kg; available Cu, 7 ± 5 mg/kg; available Pb, 5 ± 2.78 mg/kg; available Ni, 2.10 ± 1 mg/kg; coarse sand, 11%; medium sand, 42%; very fine sand, 40%; clay, 1%; silt, 6%; and sand, 21.4%.

### Determination of hydrocarbons (PAH and TPH) in the soils

Soil chemical analyses were performed by ALS Finland Oy and SGS Oy (Helsinki, Finland). Soil pH, total carbon (C), and organic C, as well as total nitrogen (N), were measured according to the ISO 10390, ISO 10694, and ISO 13878 standards, respectively. Polycyclic aromatic hydrocarbons and TPH were analyzed using n-hexane/acetone (1:1) extraction according to the methods EPA 8270, EPA 8131, EPA 8091, ISO EN 9377-2, ISO 16703, ISO 18287, and ISO 6468, as described in Mukherjee et al. (2014). We used chromatography and mass spectrometry (GC–MS) to determine hydrocarbon concentrations in the soil. Briefly, hydrocarbons in the extracts were quantified by GC–MS in a selected ion monitoring mode. The GC–MS system consists of an Agilent 6890 N gas chromatograph, coupled to a mass spectrometer detector (5975C).

### Hydrocarbon removal percentage

Pollutant removal percentage was calculated using the following expression described by Sivaram et al. (2018):

$$ Pollutants\ removal\ \left(\%\right)=\frac{100\times \left(\mathrm{CI}-\mathrm{CT}\right)}{\mathrm{CI}} $$

where:

CI: is the initial concentration of the pollutants in the soil before phytoremediation.
CT: is the final concentration of the pollutants in the soil after phytoremediation.

## Statistical analysis

The SPSS (version 25.0, IBM Corporation, Armonk, NY, USA) and Microsoft Excel 2016 (Redmond, WA, USA) were used for statistical analysis. The analysis of variance (ANOVA) with multiple comparisons of means was analyzed by the Tukey LSD test to determine the statistical difference in height among the clones at *p* < 0.05. The multivariate analysis of variance (MANOVA) was run to examine the effect of blocks and clones on pollutant removal. The pairwise *t* test was run to examine the differences in pollutant removal between 2011 and 2017.

## Results

### Survival rate and mean height growth of European aspen and hybrid aspen clones

**Fig. 2:**
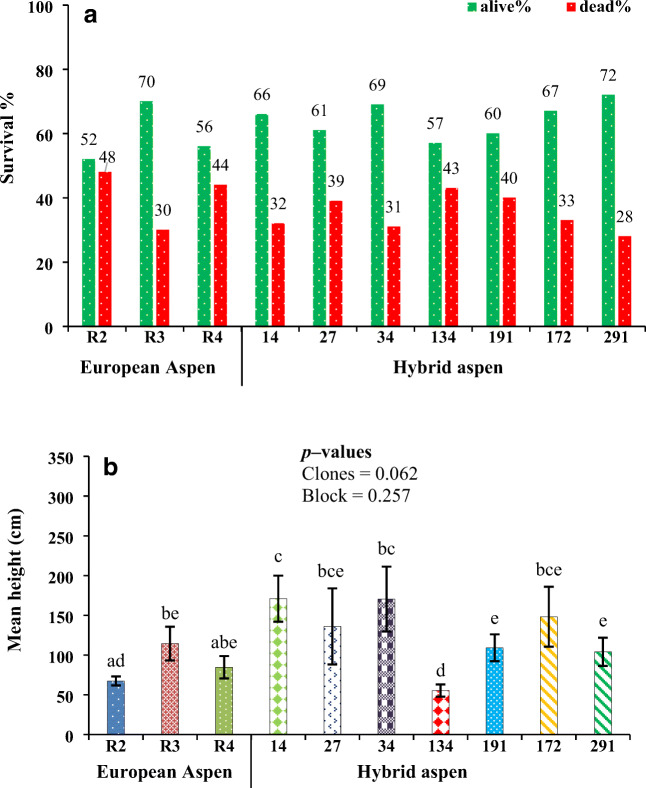
Growth of European aspen and hybrid aspen, (A) survival percentage, and (B) absolute mean height of clones grown in polluted soils. The data indicate mean values ± SE (standard error) (*n* = 4). Similar lowercase letters above the bars indicate no significant differences between clones (*p* > 0.05). Error bars show the 95% confidence interval

The mean height and survival rates for European aspen and hybrid aspen clones in the polluted plots are shown in Fig. 2A and B. During the growth period, the average survival rate was 59% for European aspen and 64% for hybrid aspen, which indicates that all the clones could survive in polluted soils (Fig. 2A). No visual symptoms of pollutant toxicity (wilting or discoloration) were observed for European aspen and hybrid aspen clones. The highest (72 %) and lowest (52 %) survival rates were observed in clones 291 and R2, respectively. Hybrid aspen clones 14 and 34 exhibited the highest height growth (171 cm) among all the studied clones. In the European aspen clones, R3 produced the greatest height growth (114 cm), followed by clones R4 and R2, respectively. The lowest height growth rate was observed in clone 134 as compared with the other hybrid aspen clones (Fig. 2B). Moreover, when exposed to fertilization, height and survival rate in clone R3 increased in comparison with clones R2 and R4. When the hybrid aspen clones 134 and 291 were subjected to fertilization, the latter displayed better height growth and survival rate than the former. Differences in mean height between the studied clones were not consistent. However, there was a slight non-significant difference among the clones for height growth, and a non-significant difference was observed between blocks I and IV (Fig. 2B).

### TPH and PAH removal by European aspen and hybrid aspen clones

To estimate PAH (mean of sixteen priority pollutants) and TPH removal percentage, soil samples were collected at two depths (5–10 cm and 10–50 cm) for every clone (Tables 1 and 2). Therefore, we present a comparison among the clones and between depths in terms of pollutant removal. Initial TPH and PAH levels in the polluted plots were higher than the final levels after phytoremediation, but some of the plots did not show a reduction in the concentration of certain pollutants. In this study, PAH and TPH (C_22_–C_40_, C_10_–C_40_, and C_10_–C_21_) concentrations at 5–10 cm depth were reduced by clone R2 in plot G15 by approximately 86 and 82%, respectively. At 10–50 cm depth, the removal percentage was 90% for PAH, 84% for C_22_–C_40_, 81% for C_10_–C_40_, and 71% for C_10_–C_21_. The highest removal rate was observed in plot I17, planted with clone 134: 97% for C_22_–C_40_, 96% for C_10_–C_40_, 90% for C_10_–C_21_, and 52% for PAH at a depth of 5–10 cm (Table 1). At a depth of 10–50 cm, no C_10_–C_21_, C_10_–C_40_, and PAH were removed, and there is only 20% of C_22_–C_40_ (Table 2). In addition, no removals occurred in the fertilized plots H14 and H15, planted with clones R3 and 291, respectively. In contrast, 74% C_22_–C_40_, 70% C_10_–C_40_, 62% PAH, and 52% C_10_–C_21_ were removed in plot H13, planted with clone 134 (with fertilizer) at 5–10 cm depth. At 10–50 cm depth, no reduction was observed for C_10_–C_21_ (increased by 207%) and C_10_–C_40_ (increased by 5%), although a 20 and 4% decrease were observed for C_22_–C_40_ and PAH, respectively.

**Table 1:** Pollutants’ concentration (mg/kg) and their removal percentage at 5–10 cm depth by European aspen and hybrid aspen clones in 2011 and 2017

Plot	pH	Year	C_10_–C_21_	C_22_–C_40_	C_10_–C_40_	PAH
G11	7	2011	152	455	606	161
		2017	180	280	474	190
		Removal %	− 18	38	22	- 18
G12	7.7	2011	494	843	1340	218
		2017	240	510	750	290
		Removal %	51	44	43	− 33
G13	6.6	2011	265	524	789	488
		2017	140	280	420	150
		Removal %	47	47	47	69
G14	6.9	2011	176	1022	735	215
		2017	290	630	920	265
		Removal %	− 65	38	− 25	− 23
G15	5.7	2011	570	1780	2350	471
		2017	100	320	420	65
		Removal %	82	82	82	86
H11	6.6	2011	174	747	40	481
		2017	54	120	170	42
		Removal %	68	83	− 325	91
H12	6	2011	173	1080	1250	179
		2017	170	370	540	140
		Removal %	2	66	57	22
H13	5.7	2011	332	1530	1860	422
		2017	160	400	560	170
		Removal %	52	73	70	62
H14	6.1	2011	556	2120	2675	532
		2017	1090	1450	2550	1050
		Removal %	− 96	32	5	− 97
H15	5.5	2011	370	1700	2070	714
		2017	340	800	1100	310
		Removal %	8	53	47	57
H16	5.4	2011	290	930	1220	180
		2017	62	140	210	160
		Removal %	79	85	83	11
H17	5.5	2011	64	330	394	34
		2017	25.5	82	104.5	19.35
		Removal %	60	75	73	43
H18	5.7	2011	30	180	210	16
		2017	20	50	61	25
		Removal %	33	72	71	− 56
I11	6.5	2011	12	106	118	6.7
		2017	20	20	40	7.7
		Removal %	− 67	81	66	− 15
I12	6.5	2011	17	73	90	15.5
		2017	20	25	40	5,4
		Removal %	− 18	66	56	65
I13	6.6	2011	16	159	174	9.87
		2017	44	100	140	42
		Removal %	− 175	37	20	− 326
I14	6.4	2011	41	191	232	39.9
		2017	58	130	190	36
		Removal %	− 41	32	18	7
I15	6.3	2011	48	278	326	89.6
		2017	260	270	530	210
		Removal %	− 442	3	− 63	− 134
I16	5.6	2011	81	260	341	43
		2017	81	140	220	220
		Removal %	0	46	35	− 412
I17	5.5	2011	200	740	940	96
		2017	20	24	40	46
		Removal %	90	97	96	52
I18	5.4	2011	350	1200	1550	160
		2017	78	150	220	290
		Removal %	78	88	86	− 81
J16	5.8	2011	20	25	40	3
		2017	21	20	40	5.05
		Removal %	− 5	20	0	− 68
J17	5.8	2011	37	180	217	18
		2017	20	41	55	11
		Removal %	46	77	75	39
J18	5.8	2011	20	85	105	8,60
		2017	20	20	40	3.70
		Removal%	0	76	62	56

**Table 2:** Pollutants’ concentration (mg/kg) and their removal percentage at 10–50 cm depth by European aspen and hybrid aspen clones in 2011 and 2017

Plot	Year	C_10_–C_21_	C_22_–C_40_	C_10_–C_40_	PAH
G11	2011	75	324	399	30
	2017	48	110	160	42
	Removal %	36	66	60	− 40
G12	2011	1620	954	2580	202
	2017	160	420	580	150
	Removal %	90	56	78	26
G13	2011	455	567	1020	428
	2017	280	220	500	350
	Removal %	38	61	51	18
G14	2011	179.5	605	433	179
	2017	290	635	915	205
	Removal %	− 62	− 5	− 111	− 15
G15	2011	293.5	887	1180	124
	2017	84	140	225	12.5
	Removal %	71	84	81	90
H11	2011	42	200	40	16,7
	2017	23	35	58	19
	Removal %	45	83	− 45	− 14
H12	2011	88	348	436	122
	2017	270	450	720	270
	Removal %	− 207	− 29	− 65	− 121
H13	2011	212	835	1050	436
	2017	400	670	1100	420
	Removal %	− 89	20	− 5	4
H14	2011	189.5	688	875.5	164
	2017	440	617.5	1075	435
	Removal %	− 93	10	− 23	− 165
H15	2011	292	1020	1310	312
	2017	450	1100	1600	440
	Removal %	− 54	− 8	− 22	− 41
H16	2011	230	960	1190	84
	2017	49	71	120	130
	Removal %	79	93	90	− 55
H17	2011	91	280	371	53
	2017	20	44	59	6.7
	Removal %	78	84	84	87
H18	2011	47	270	317	23
	2017	20	40	48	3
	Removal %	57	85	85	87
I11	2011	10	28	40	0.2
	2017	20	20	40	3
	Removal %	− 100	29	0	− 1400
I12	2011	100	57	156	0.97
	2017	61	20	81	3
	Removal %	39	65	48	− 208
I13	2011	10	21	24	1.91
	2017	20	20	40	3.6
	Removal %	− 100	5	-67	− 88
I14	2011	10	27	40	3.54
	2017	20	26	45	8.8
	Removal%	− 100	4	− 13	− 149
I15	2011	10	74	84	24.8
	2017	20	26.5	41	10.7
	Removal %	− 100	64	51	57
I16	2011	20	20	40	3
	2017	20	20	40	3
	Removal %	0	0	0	0
I17	2011	20	25	40	3.8
	2017	20	20	40	5
	Removal %	0	20	0	− 93
I18	2011	110	450	560	44
	2017	36	81	120	33
	Removal %	67	82	79	25
J16	2011	20	36	40	3
	2017	25	20	40	3
	Removal %	-25	44	0	0
J17	2011	20	20	40	3
	2017	20	20	40	0.13
	Removal %	0	0	0	90
J18	2011	20	20	40	3
	2017	20	23	40	3
	Removal %	0	− 15	0	0

### Total mean removal rate of PAH and TPH from blocks I to III

**Fig. 3:**
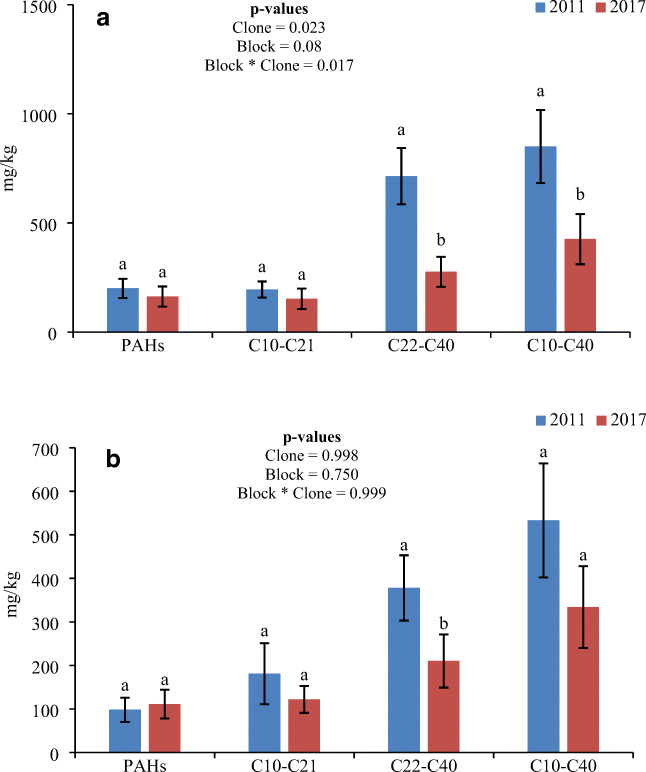
Total mean removal of PAH and TPH concentrations from three blocks: (A) depth 5–10 cm; (B) depth 10–50 cm. The data indicate the means ± SE (standard error) (*n* = 4). Similar lowercase letters above the bars indicate no significant differences (*p* > 0.05) between years. Error bars show the 95% confidence interval

The total mean removal rate of PAH, C_10_–C_21_, C_22_–C_40_, and C_10_–C_40_ from blocks I–III at a depth of 5–10 cm was 18, 22, 61, and 50 %, respectively (Fig. 3A). A similar trend at 10–50 cm depth was observed, for example, C_10_–C_21_ (32 %), C_22_–C_40_ (44 %), and C_10_–C_40_ (37 %), while PAH concentration increased by approximately 13% (Fig. 3B). A significant difference was observed among the clones in the soil samples collected at a depth of 5–10 cm, but no significant differences between the blocks were found (Fig. 3A). Non-significant differences among the clones and blocks were seen when the pollutant level was measured in the soil samples collected at a depth of 10–50 cm (Fig. 3B). Greater pollutant removal was observed for TPH compared to PAH. Moreover, the greatest pollutant removals were found at a depth of 5–10 cm compared with 10–50 cm. A significant reduction in PAH was only observed in the 5–10 cm soil zone. However, the degradation of PAH and TPH was most pronounced in the upper soil surface (5–10 cm) and decreased with increasing depth.

## Discussion

The present study investigates the phytoremediation potential of three European aspen and seven hybrid aspen clones in polluted petroleum soils. During the experiment, the aspen plants grown in polluted soils did not exhibit stress symptoms (by visual observations). However, some of the clones died, which may have resulted from specific environmental factors, such as planting methods, excessive or insufficient rainfall, disease/pests, weed competition, and contamination (Nichols et al. 2014). However, plant growth may have been affected and these plants may not have been able to tolerate the concentration of hydrocarbons in the soil (Peng et al. 2009). According to Anyasi and Atagana (2018), when plants are grown in hydrocarbon-polluted soils, they generally experience some metabolic fluctuations, for example, hormonal production and enzyme sequestration, which brings about substrate modification and boosts the activities of the enzymes.

Nguemté et al. (2018) and Shirdam et al. (2008) reported that the inhibition of plant growth can also be caused by a toxic compound in TPH, especially the low-molecular-weight PAH. In some cases, site quality differences are most likely driven by nutrient concentration, physical characteristics, and water holding capacity of the soil, while differences might also be caused by climatic variables, e.g., growing degree days (Guidi Nissim et al. 2018). Moreover, petroleum hydrocarbons in contact with the soil form an impermeable coating at the surface, which prevents water circulation in the soil and gas exchange between the soil and air, resulting in the suffocation of the plant roots. As the soil becomes anaerobic, metabolic activity and the number of aerobic bacteria then decrease (Streche et al. 2018).

The highest pollutant removal rates were observed in hybrid aspen clones 134 and R2. Overall, all the clones showed adequate removal rates, but a variation was seen across different plots at the two depth levels. This could be caused by many factors; for example, the plots exhibited different pollutants and pH levels, the action of root exudates (which aid in improving the bioavailability of PAH to the plants), the plant-specific rhizospheric effect, and differences in the inherent nature of the plants. Tischer and Hübner (2002) found that the highest reduction (64%) of hydrocarbon concentrations occurred in the subsoil layer rather than in the deep soil layers, as did Vervaeke et al. (2003), which is in agreement with our results. Nevertheless, PAH were found in extremely low concentrations in our soil samples, which suggests their degradation, evaporation, or transportation from the topsoil (Mukherjee et al. 2014).

According to Akinola and Njoku (2007), differences in the genetic makeup of plants produce a divergent response to contamination. Hence, the differences noted in our study can be attributed to the genetic variances among the European aspen and hybrid aspen clones. Similar findings were reported by Sivaram et al. (2018), who compared C3 and C4 plants for the remediation of PAH-polluted soils and stated that the remediation of PAH-polluted soils not only depends on the physicochemical property of PAH but also depends on the nature of the plants and the biologically available concentration of PAH (Reid et al. 2000; Sivaram et al. 2018). Our results regarding PAH degradation are in agreement with the assumption of Noori et al. (2018), who documented that the plant roots can facilitate the degradation of pollutants in the soil. They also suggested that PAH degradation could also be the result of plant enzymatic and biochemical activities, as well as the enhancement of microbial community activity in the rhizosphere. In addition, PAH degradation in polluted soils possibly occurs because of the ability of the indigenous microbial community to acclimatize to the polluted area, which results in the degradation of the targeted pollutants (García–Delgado et al. 2019; Lukic et al. 2016).

Soil pH plays a vital role during the biodegradation process, as well as in the growth and activity of soil microorganisms, as it regulates the solubility, mobility, and the availability of the ionized forms of pollutants (Kalita and Devi 2012). According to Kalita and Devi (2012), significant degradation of petroleum hydrocarbons commonly occurs between pH 4.5 and 7.5, and the pH of the soil in our study falls within this range. As such, the pH levels in our soils were favorable for the degradation of TPH and PAH and also for their efficient removal from the soil. Our results have demonstrated that variation in soil pH (between different plots) may also affect plant growth and the efficient removal of pollutants by the plant. Previously, Mukherjee et al. (2014) reported the critical role of pH at the same study site (in Luumaki) and demonstrated its importance as an environmental variable that can regulate bacterial communities in the soil. Moreover, the pH gradient permits the development of considerable niche differentiation. At lower pH levels, Burkholderiacea and Acetobacteraceae predominate, while Pseudomonadaeceae and Sphingomonadaceae are found at higher pH levels. Silby et al. (2011) have reported that Burkholderiacea, Sphingomonadaceae, and Pseudomonadaeceae are known for their catabolic flexibility and are also considered the most common hydrocarbon degraders in a range of soils. Our study highlights the pH preferences of these bacterial groups, and the variances observed in the degradation of the studied organic pollutants might advocate that different microbiological processes were accountable for the deterioration of PAH and TPH.

Nutrients, such as N, P, and K, are very important constituents for plant growth development and can also support the successful biodegradation of hydrocarbons. Some of these nutrients could become a limiting factor at elevated levels, thus affecting the biodegradation processes and inhibit biodegradation activity (Das and Chandran 2011). Many authors have described the conflicting effects of high NPK levels on the biodegradation of hydrocarbons, particularly on aromatics (Chaîneau et al. 2005; Das and Chandran 2011), which is consistent with the findings in some of the plots in our study (H13, H14, and H15) where no hydrocarbon removal was observed between years. This could be due to the high concentration of nutrients, which interrupted the degradation process by affecting the microbial community in the rhizosphere. Moreover, this inhibition of hydrocarbon degradation in fertilized plots may be the result of a reduction in nutrient levels due to the acclimatization of other C sources in the soil and could also be ascribed to the non-invigoration of the aromatic degrading microorganisms (Carmichael and Pfaender 1997; Chaîneau et al. 2005).

Afforestation using multiple clones has been shown to strongly increase the efficiency of phytoremediation (Wei and Pan 2010). Moreover, the degradation of organic pollutants by multiclonal plantation may consequently stimulate the degradation rate of some intransigent organic pollutants, microbial functional diversity, higher soil microbial biomass, and the deterioration of enzyme activities (Chen et al. 2013). However, our results have demonstrated that this study may provide valuable information for the establishment of phytoremediation experiments in regard to the rehabilitation of polluted petroleum soils with European aspen and hybrid aspen clones.

## Conclusions

We assume that the phytoremediation potential is proportional to plant survival and growth since successful tree establishment is the first requirement for long-term petroleum degradation in the soil. Moreover, tree planting is not only limited to phytoremediation but could provide critical ecological services that will positively contribute to cost-effectiveness and social perception. In this context, we have demonstrated that these European aspen clones are able to establish on polluted hydrocarbon soils. All the studied clones survived well in the polluted environment, and the survival rate of most clones was above 50%; European aspen clone R3 (70%) and hybrid aspen clone 291 (72%) exhibited the highest survival rates. Hybrid aspen clones 14, 34, and 172 produced the largest height growth among the studied clones, with the least height growth observed in clone 134. All clones displayed good potential in terms of pollutant removal but clones R2 and 134 performed best. In the experimental area (~ 2 ha), the efficiency of the clones to reduce pollutant levels was only assessed in the severely polluted plots from blocks I to III, yet the survival and height growth rates of the plants were measured from all the plots of blocks I–IV. However, our results should be regarded as preliminary, as the field trial lacked sufficient replications and experimental controls, which suggests further investigation is needed. To provide more insight, additional research should be conducted in regard to the mechanisms of hydrocarbon degradation, inoculation with different bacteria, fertilizer application rates, and the addition of organic chelates in European aspen and hybrid aspen plantations.
